# Relationship between thyroid-stimulating hormone, BDNF levels, and hippocampal volume in antipsychotic-naïve first-episode psychosis patients

**DOI:** 10.3389/fpsyt.2023.1301714

**Published:** 2023-12-07

**Authors:** Alba Toll, Laura Blanco-Hinojo, Daniel Berge, Ana Manzano, Khadija El Abidi, Víctor Perez-Solà, Anna Mané

**Affiliations:** ^1^Institut de Salud Mental, Hospital del Mar, Barcelona, Spain; ^2^Hospital del Mar Research Institute, Barcelona, Spain; ^3^Centro de Investigación Biomédica en Red, Área de Salud Mental (CIBERSAM), Madrid, Spain; ^4^MRI Research Unit, Department of Radiology, Hospital del Mar, Barcelona, Spain; ^5^Department of Medicine and Life Sciences, Universitat Pompeu Fabra (UPF), Barcelona, Spain

**Keywords:** thyroid hormone, BDNF (brain derived neurotrophic factor), TSH (thyroid stimulating hormone), hippocampus, hippocampal volume, drug-naïve, first episode psychosis (FEP)

## Abstract

**Introduction:**

Thyroid hormones play an essential role in hippocampal development, a key structure in psychosis. However, the role of these hormones in first-episode psychosis (FEP) has received limited attention. It has been hypothesized that thyroid hormones could cause morphological modifications in the hippocampal structure through the upregulation of brain-derived neurotrophic factor (BDNF). In this study, we primarily aimed to determine the relationship between thyroid-stimulating hormone (TSH) levels, peripheral BDNF levels, and hippocampal volume in antipsychotic-naïve FEP patients. We also aimed to determine whether TSH levels were associated with clinical symptomatology.

**Materials and methods:**

A total of 50 antipsychotic-naïve FEP patients were included in the study. At baseline, we collected fasting blood samples and registered sociodemographic and clinical variables (substance use, DUP, PANSS, GAF, and CDSS). Structural T1 MRI was performed at baseline to quantify brain volumes. No control group was used for this study.

**Results:**

Of the 50 patients, more than one-third (36%) presented alterations in TSH levels, mainly elevated levels (32% of patients). The TSH levels were inversely correlated with both peripheral BDNF and hippocampal volume. On the multivariate analysis, the model that best predicted the relative hippocampal volume was a single variable model (TSH levels). No significant association was observed between TSH levels and clinical symptomatology.

**Discussion:**

These results suggest that thyroid hormones could have a neuroprotective effect on the hippocampus in FEP patients, possibly through their effect by increasing BDNF concentrations, which could attenuate brain injury and neuroinflammation. Nevertheless, thyroid hormones could also affect hippocampal volume through other pathways.

## Introduction

The role of alterations in thyroid hormones has received limited attention in schizophrenia and first-episode psychosis (FEP). However, these hormones play an essential role in brain development (1), especially in the hippocampus. Structural and functional changes in the limbic system and the medial temporal lobe are central to the symptomatology associated with psychosis (2), and smaller relative hippocampal volumes are commonly observed in patients with schizophrenia and FEP (3). Furthermore, these hippocampal abnormalities are associated with the emergence of psychotic symptomatology in patients with schizophrenia and FEP (4, 5).

Deficiencies in these hormones during the neonatal period have been associated with deleterious effects on neural growth and development, including reduced synaptic connectivity, delayed myelination, disturbed neuronal migration, and deranged axonal projections, especially in the hippocampus (6). Thyroid hormones also interact with glial cells in the adult brain, thereby modulating immune response, regulating neurotransmitters (dopamine, serotonin, glutamate, and GABA), and controlling neuron metabolism (7). Taking all the above characteristics into account, some authors have suggested that thyroid hormones could play a role in the pathophysiology of psychosis (8, 9).

Thyroid-stimulating hormone (TSH) is a glycoprotein pituitary hormone that stimulates the thyroid gland to produce thyroxine (T4) and then triiodothyronine (T3), which stimulates the metabolism of almost every tissue in the body (7). The findings of a recent meta-analysis (10) suggested that TSH levels might be decreased in FEP patients and elevated in patients with multiple-episode schizophrenia. The authors of the meta-analysis suggested that these alterations could be attributable to the effects of increased dopaminergic activity on the secretory functions of the pituitary gland, which in turn can alter TSH levels. Other authors have suggested that higher baseline TSH levels could be associated with poorer treatment response in patients with schizophrenia (11). Nevertheless, only a few studies have assessed the relationship between thyroid hormones and clinical features of schizophrenia and FEP, with inconclusive results (9, 12). Furthermore, most of the studies carried out to date have involved patients on antipsychotic medications, which could affect thyroxine levels (13). Consequently, it would be interesting to further explore this potential relationship in antipsychotic-naïve FEP patients to clarify this issue.

While thyroid hormones play a key role in brain function, other factors such as brain-derived neurotrophic factor (BDNF) are also important. BDNF is essential for neuronal survival and network plasticity. Moreover, it is the most widely distributed neurotrophin in the central nervous system (CNS), with an especially high concentration (similar to thyroid receptors) of neurotrophins in the hippocampus (14). Interestingly, peripheral BDNF levels strongly correlate with brain BDNF levels, which allow their use as a peripheral biomarker (15). BDNF is also involved in the etiopathogenesis of several psychiatric disorders (16, 17), including psychotic disorders (18). Furthermore, an association between BDNF and hippocampal volume in schizophrenia and FEP has been well documented (19).

Animal models have shown that rats with early-onset hypothyroidism have low BDNF levels (20). In human, one study involving patients with depression found that higher TSH levels were associated with lower serum BDNF levels and a smaller increase in BDNF during antidepressive treatment (21). It has been postulated that thyroid hormones could cause morphological modifications in the hippocampal structure through the upregulation of BDNF mRNA expression and, therefore, induce a significant increase in BDNF protein levels in this brain structure (22). However, the exact mechanism that would explain the potential association between thyroid hormone and BDNF levels in schizophrenia and FEP has received limited attention. In fact, to our knowledge, the association between TSH and BDNF levels in FEP has not been assessed to date.

In this context, the present study primarily aimed to determine and assess the relationship between peripheral BDNF levels, TSH levels, and hippocampal volume in a series of antipsychotic-naïve FEP patients (no healthy controls). The study also aimed to determine the association between TSH levels and clinical symptomatology in these patients. We hypothesized the following: (1) antipsychotic-naïve FEP patients would have altered TSH levels; (2) TSH levels would be associated with clinical symptomatology in these patients; and (3) higher TSH levels would be associated with lower peripheral BDNF levels and lower hippocampal volume in these patients.

## Materials and methods

### Study population

A total of 50 antipsychotic-naïve FEP patients treated under the Estudi i Tractament d’Episodis Psicòtics (ETEP) program of the Study and Treatment Program for First-Episode Psychosis at the Hospital del Mar, Barcelona, Spain between April 2013 and July 2017 were consecutively included in this study. The ETEP program is a specialized early intervention service for individuals aged between 18 and 35 years diagnosed with FEP. The program offers a multimodal intervention, including comprehensive assessment and intensive medical and psychosocial treatments (see Supplementary material for details).

The inclusion criteria were as follows: (1) patients aged between 18 and 35 years; (2) those that fulfilled the DSM-IV-TR criteria for any of the following: brief psychotic disorder, schizophreniform disorder, schizophrenia with <1 year of symptoms, or unspecified psychosis; (3) those with no prior history of severe neurological medical conditions or severe traumatic brain injury; (4) those with presumed IQ level > 80 based on clinical records; and (5) those with no substance abuse or dependence disorders except for cannabis and/or nicotine use. All patients were affected by antipsychotic- and antidepressant-naïve disorders. They were treated with benzodiazepines, and no control group was used for this study.

This study was approved by the local ethics committee at Hospital del Mar, Barcelona, Spain (2021/10093). All participants provided written informed consent. The study protocol complies with all ethics criteria established by the Declaration of Helsinki and with all local laws on patient confidentiality and data protection.

### Clinical assessment and demographic data

At baseline, all patients underwent a comprehensive evaluation performed by two experienced psychiatrists (A.M. and D.B.). Sociodemographic variables (age and sex) were recorded. Substance use was assessed, including tobacco (cigarettes per day) and cannabis use (“joints” per week, after dichotomization between users and non-users). The Structured Clinical Interview for DSM-IV-TR Axis I Disorders was performed to make the diagnosis. The following scales were administered: the Positive and Negative Syndrome Scale (PANSS) for symptoms related to psychosis (23); the Global Assessment of Functioning (GAF) (24) for functionality; and the Calgary Depression Scale for Schizophrenia (CDSS) for depressive symptoms (25).

### Collection of blood samples and determination of serum BDNF levels in patients at baseline

Fasting blood samples were obtained upon arrival at the health center (i.e., at baseline) before administering any medication (except for benzodiazepines). All blood samples were obtained in the morning (between 8 a.m. and 12 p.m.) to avoid circadian-related fluctuations in BDNF levels, which have been reported to occur in men but not in women (26). Blood samples were collected in glass K3–EDTA blood-drawing tubes for whole blood. The serum was isolated by centrifugation at 300*x* g for 15 min, removed, and stored at – 80°C until analysis.

BDNF levels were measured using the ChemiKine Sandwich ELISA kit (Chemicon, Temecula, California; United States) according to the manufacturer’s instructions. The Wallac Victor 2 microplate reader (wavelength, 450 nm) was used to determine absorbance. BDNF concentrations were determined according to the standard curve, which was constructed from duplicate samples containing appropriate concentrations. All samples were analyzed in duplicate. The calculated overall intra- and inter-assay variation coefficients were 3.7 and 8.5%, respectively; the detection limit of the BDNF assay was 15 pg./mL.

TSH levels were determined under routine conditions on the same day of the blood draw. The hormonal assay was performed at the testing laboratory of the Hospital del Mar, Barcelona, Spain. TSH was determined by electrochemiluminescence immunoassay, and levels between 0.30 and 4.20 mcUI/mL were considered normal for both sexes. TSH inter- and intra-assay coefficients of variability were < 5%.

### Image acquisition and processing

Baseline brain imaging was performed with a Philips Achieva 3.0 Tesla magnetic resonance imaging (MRI) scanner (Philips Healthcare; Best, Netherlands) equipped with an eight-channel phased-array head coil. The imaging protocol involved the acquisition of high-resolution anatomic three-dimensional (3D) images based on a T1-weighted fast spoiled gradient inversion recovery prepared sequence with the following parameters: repetition time, 8.2 ms; echo time, 3.8 ms; flip angle, 8°; field of view, 24 cm; pixel matrix, 256 × 256; in-plane resolution, 0.94 × 0.94 mm^2^; and slice thickness, 1 mm.

Anatomical images were visually inspected before analysis by a trained operator to detect any motion effect. No gross brain abnormalities were identified. The quality of the raw T1-weighted MRI scans was quantitatively assessed by using the automated weighted image quality rating (IQR), a measure of general image quality combining parameters of noise, inhomogeneities, and spatial resolution, provided by the CAT12 toolbox.^1^ Scores >60% were considered to be of sufficient quality for inclusion in subsequent analyses; all of the scans met this criterion.

Volumetric segmentation was performed with the fully automated and validated segmentation software package FreeSurfer v6.0^2^ with the default “recon-all” stream, as described in previous studies (27–29). Briefly, important preprocessing steps included the removal of non-brain tissue, intensity normalization, automated Talairach transformation, and segmentation of the subcortical white matter and deep gray matter volumetric structures (30). Segmentations of the hippocampus (left and right hemisphere) were visually inspected for accuracy by overlaying the segmentation label of each structure on the individual T1-weighted brain scan following the ENIGMA consortium’s imaging quality control protocol for subcortical segmentations.^3^ The estimated hippocampal volumes of the left and right hemisphere and estimated total intracranial volume were extracted for each participant from the aseg.stats output files in FreeSurfer.^4^ Group-level means and standard deviations and histogram plots were used to identify statistical outliers (defined as measured volumes >2.698 standard deviations [SD] from the global mean) and to confirm the normal distribution of volumetric data.

The relative volume ratio for the anatomical regions of interest was calculated as the volume in native space (ml)/total intracranial volume (L). The MRI assessment was performed when patients were able to stand still (not having high psychomotor agitation or disorganized behavior to tolerate the MRI procedure) but within a 2 weeks window from the initial assessment.

### Statistical analysis

Data distribution normality was assessed with the Kolmogorov–Smirnov test. A descriptive analysis of the sample was performed, including the percentage of patients with TSH alterations. We then evaluated the differences between sociodemographic, clinical, and neurobiological variables between patients based on their thyroid status (hypothyroidism, hyperthyroidism, or normal thyroid function). We evaluated the correlation (Pearson’s test) between TSH levels and clinical symptomatology (PANSS total score and subscale scores, CDSS, and GAF). Next, we determined the correlation (Pearson’s test) between TSH and BDNF levels and between TSH levels and relative hippocampal volume. Finally, a linear regression model (step-wise method) was performed using relative hippocampal volume as the dependent variable. Based on previous publications (29, 31), we included the following independent variables: age, sex, duration of untreated psychosis (DUP), nicotine use, cannabis use, TSH levels, and BDNF levels.

All statistical analyses were performed with the IBM-SPSS Statistics for Windows, v. 20 (IBM Corp.; Armonk, NY, United States). *p*-values of ≤0.05 were considered statistically significant.

## Results

### Characteristics of FEP patients

A total of 50 patients were included in this study. Most of the patients (*n* = 30; 60%) belonged to male sex. The median (IQR) age was 26 (24–30.25) years. The mean (SD) TSH level was 2.83 (2.21) mcIU/mL, and the mean (SD) BDNF level was 45.27 (27.14) ng/mL. The mean (SD) left and right absolute hippocampal volumes were, respectively, 3.88 (± 0.38) and 4.09 (± 0.45), which are comparable to the volumes previously reported in similar clinical populations using the same FreeSurfer method (19–21).

The other sociodemographic, clinical, biochemical, and volumetric characteristics of the patients at baseline are shown in Table 1.

**Table 1:** Sociodemographic, clinical, and neurobiological variables in FEP patients.

Variable	Patients (*N* = 50)
Age, median (IQR)	26 (24–30.25)
Female sex, *n* (%)	20 (40)
DUP, median (IQR)	31 (8–115)
Cannabis use, *n* (% users)	29 (58)
Tobacco use, median (IQR)	4.5 (0–14)
BDNF levels in ng/mL, m (SD)	45.27 (27.14)
TSH levels in mcIU/mL, m (SD)	2.83 (2.21)
PANSS P score, m (SD)	24.88 (6.74)
PANSS N score, m (SD)	16.86 (6.65)
PANSS GP score, m (SD)	43.68 (8.27)
PANSS T score, m (SD)	85.24 (15.76)
CDSS score, m (SD)	1.22 (2.02)
GAF score, m (SD)	29.7 (8.89)
Relative total hippocampus volume in mL	3.98 (0.42)

*N*, sample size; IQR, interquartile range; m, mean; SD, standard deviation; DUP, duration untreated psychosis; BDNF, brain-derived neurotrophic factor; TSH, thyroid-stimulating hormone; PANSS P, Positive and Negative Syndrome Scale positive; PANSS N, Positive and Negative Syndrome Scale negative; PANSS GP, Positive and Negative Syndrome Scale general pathology; PANSS T, Positive and Negative Syndrome Scale total; CDSS, Calgary Depression Scale for Schizophrenia; GAF, Global Assessment of Functioning; mL, milliliter.

### TSH levels and clinical symptomatology in FEP patients

In this 50-patient sample, TSH levels were classified as follows: normal (*n* = 32; 64%), high (*n* = 16; 32%), and low (*n* = 2; 4%). There were no significant differences between the three groups (hypothyroidism, hyperthyroidism, and normal thyroid function) in terms of sociodemographic or clinical variables (Table 2). No significant correlations between TSH levels and clinical symptomatology were observed (Table 3).

**Table 2:** Differences in sociodemographic, clinical and neurobiological variables between groups (hypothyroidism, normal thyroid function, and hyperthyroidism) in FEP patients.

Variable	Hypothyroid (32%)	Normal thyroid function (64%)	Hyperthyroid (4%)	*p*
Age, median (IQR)	26 (22–28)	23 (20–26)	23.5 (22–24)	0.135
Female sex, *n* (%)	7 (43.8)	11 (34.4)	2 (100)	0.172
Tobacco use, median (IQR)	5.5 (0.25–14)	2 (0–14)	10 (5–14)	0.533
Cannabis use, *n* (% users)	12 (75)	17 (53.1)	0 (0)	0.133
DUP in days, median (IQR)	60.5 (17–143)	87.39 (8–108)	60 (50–65)	0.888
PANSS P, m (SD)	25 (8.41)	24.75 (6.12)	26 (1.41)	0.966
PANSS N, m (SD)	16.38 (7.52)	17.5 (6.23)	10.5 (3.53)	0.337
PANSS PG, m (SD)	32.4 (8.83)	32.1 (7.29)	26 (1.41)	0.679
PANSS T, m (SD)	60.53 (13.86)	64.1 (15.64)	46 (1.41)	0.873
CDSS score, m (SD)	1.63 (2.64)	1.09 (1.73)	0 (0)	0.483
GAF, m (SD)	30.88 (10.44)	29.5 (8.27)	23.5 (4.95)	0.541
BDNF in ng/mL, m (SD)	36.68 (19.46)	45.67 (26.25)	107.44 (10.67)	**0.001**
Relative total hippocampus volume, m (SD)	3.23 (0.25)	3.36 (0.66)	3.47 (0.55)	**0.047**

*N*, sample size; IQR, interquartile range; m, mean; SD, standard deviation; ng, nanogram; DUP, duration untreated psychosis; BDNF, brain-derived neurotrophic factor; PANSS P, Positive and Negative Syndrome Scale positive; PANSS N, Positive and Negative Syndrome Scale negative; PANSS GP, Positive and Negative Syndrome Scale general pathology; PANSS T, Positive and Negative Syndrome Scale total; CDSS, Calgary Depression Scale for Schizophrenia; GAF, Global Assessment of Functioning; mL, milliliters.

**Table 3:** Correlations between TSH levels and clinical symptomatology in FEP patients.

	PANSS P	PANSS N	PANSS GP	PANSS T	CDSS	GAF
Pearson’s correlation	0.08	0.07	0.24	0.11	−0.3	−0.19
*p*	0.495	0.685	0.149	0.495	0.057	0.236

PANSS P, Positive and Negative Syndrome Scale positive; PANSS *N*, Positive and Negative Syndrome Scale negative; PANSS GP, Positive and Negative Syndrome Scale general pathology; PANSS T, Positive and Negative Syndrome Scale total; CDSS, Calgary Depression Scale for schizophrenia; GAF, Global Assessment of Functioning.

### TSH levels, peripheral BDNF levels, and relative hippocampal volume in FEP patients

**Figure 1:**
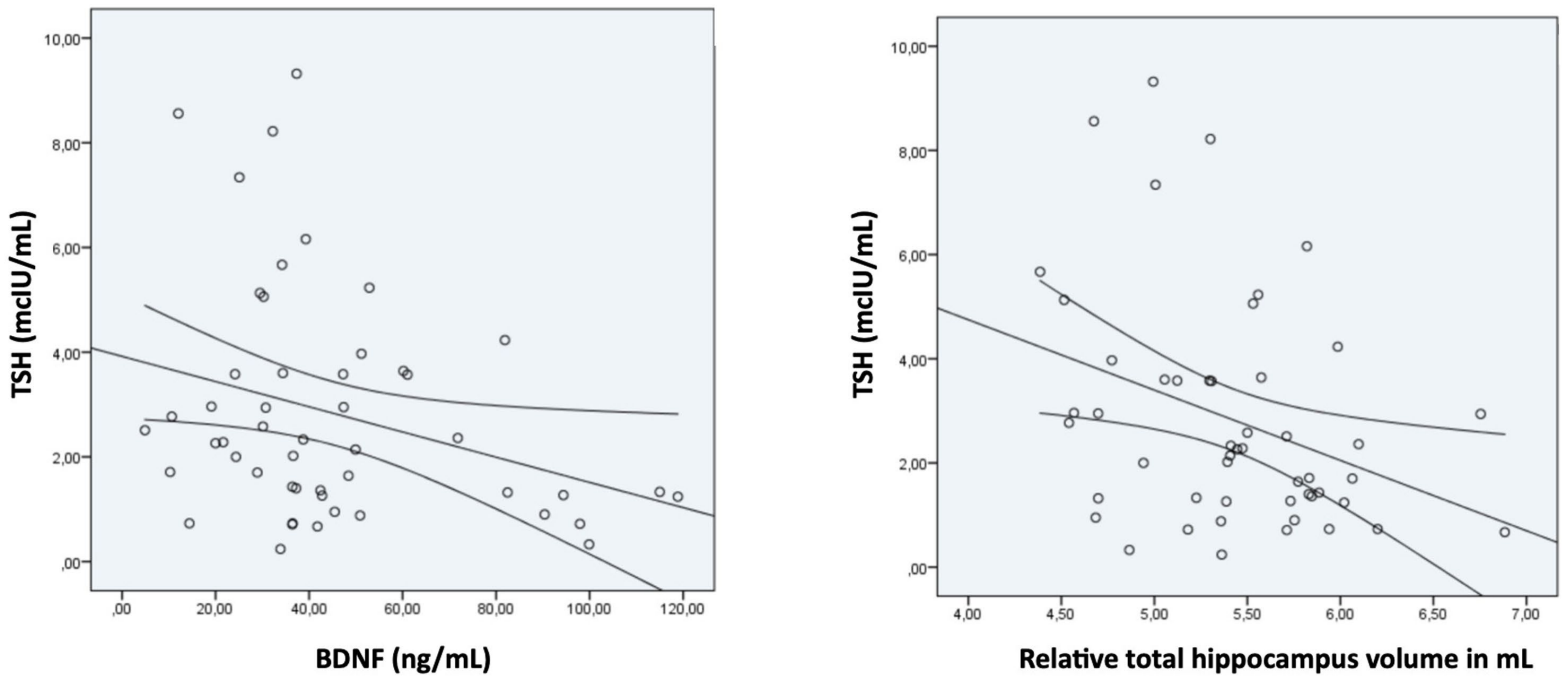
Correlation between TSH levels and BDNF levels and relative total hippocampal volume in FEP patients at baseline.

Significant between-group differences were observed in terms of BDNF levels (*p* = 0.001) and relative hippocampal volume (*p* = 0.047) (Table 2). TSH levels were negatively correlated with BDNF levels (*r* = −0.29; *p* = 0.036) and with the total relative hippocampal volume (*r* = −0.34; *p* = 0.016) (Figure 1).

On the multivariate analysis, the model that best predicted relative hippocampal volume was a single variable model (TSH levels), with an adjusted *R*^2^ of 0.345. Thus, higher TSH levels (95% confidence intervals [CIs] -0.15 to −0.02; *p* = 0.016) were significantly associated with lower relative hippocampal volume at baseline.

## Discussion

The main aim of this study was to determine the possible association between peripheral BDNF concentration, TSH level, and hippocampal volume in a group of antipsychotic-naïve FEP patients. The second aim of the study was to assess the association between TSH levels and clinical symptomatology in these patients. More than one-third (36%) of these patients had alterations in TSH levels, mainly higher levels (32% of the sample). In addition, TSH levels were significantly associated with both peripheral BDNF concentration and hippocampal volume.

Studies conducted to date to investigate thyroid hormone levels in patients with psychotic disorders have reported mixed findings. A recent meta-analysis (10) found higher TSH levels in multi-episodic schizophrenia but lower TSH levels in antipsychotic-naïve FEP patients. The reason for this discrepancy between these two patient populations is not clear. However, it is important to note that the authors of the meta-analysis were not able to determine whether the observed differences in hormone levels were clinically relevant, given that many of the studies included in the analysis did not report the proportion of participants with abnormal hormone levels. A population-based study carried out by Sharif et al. (32) found that schizophrenia was significantly more prevalent in hypothyroid patients than in healthy participants, a finding that is consistent with our results. Nevertheless, the underlying mechanisms driving alterations in thyroid hormones in drug-naïve FEP patients remain largely unknown (10).

Only a few studies have explored the relationship between clinical symptoms and alterations in thyroid hormone levels. Recently, one study in FEP patients (33) found that high T4 plasma levels (but still within the normal range) were associated with fewer prodromal symptoms, shorter DUP, greater likelihood of sudden onset psychosis, and more affective symptomatology at 1 year of follow-up. A study involving patients with early psychosis found that higher T4 levels were associated with better cognitive performance on the attention domain (12) and that the presence of antithyroid antibodies was associated with worse negative symptoms (9). In our study, the lack of a significant association between TSH levels and clinical symptomatology could be explained by the limited sample size, which was sufficient to find biological differences but not to identify clinically significant differences between the groups.

We found that TSH levels were inversely correlated with both peripheral BDNF concentration and hippocampal volume. In fact, on the multivariate analyses, the TSH level was the only significant predictor of hippocampal volume. These results are in line with some model animal studies (34–36), which hypothesized that thyroid hormones regulate neurotrophic gene expression in a developmental stage and in a specific brain region.

In a developmental hypothyroid rat model (34), hypothyroidism induced neuronal loss by the downregulation of BDNF survival signaling, increasing truncation of the p75 neurotrophin receptor. In experimental models in rats with severe prenatal hypothyroidism, pups (but not parents) exhibited reduced levels of BDNF in the hippocampal tissue (35). A study involving healthy rats (36) found that thyroxine (T4) treatment during the first week after birth increased BDNF mRNA expression and protein levels in the hippocampus. Another study involving rats exposed to hypoxic ischemia who received either T4 or placebo injections found that hippocampal BDNF levels were higher in the thyroxine-treated group (37). These findings suggest that TSH, through its impact on BDNF concentrations, may offer neuroprotection to the immature brain, thus attenuating brain injury and neuroinflammation (38). In patients with schizophrenia, a decrease in BDNF signaling has been proposed as a causal effect of neuropil retraction, leading to hippocampal reduction (39).

In line with the aforementioned findings in animal models, studies in human have also reported structural changes to the hippocampus in both congenital and adult-onset hypothyroidism. In one study, patients with congenital hypothyroidism had smaller hippocampal volumes (particularly on the left side), despite early diagnosis and treatment (40). In another study, patients with adult-onset hypothyroidism presented a significant volume reduction in the right hippocampus relative to controls (41).

Thyroid hormones could also affect hippocampal volume through other pathways. One of the main characteristics of the hypothyroid brain is poor myelination caused by impaired oligodendrocyte differentiation (42). Studies have shown that differentiated oligodendrocytes are depleted in animals with hypothyroidism, leading to a reduced amount of myelin deposition in white matter axons (43). Thyroid deficiency has also been reported to affect the expression and activity of prostaglandin D2 synthase, which may alter key brain processes such as myelination and other neuromodulating functions in the CNS (44).

### Strengths and limitations

This study has several limitations, and the most notable one is the observational design, which does not allow us to infer a cause–effect relationship. Although the main aim of the study was to determine and assess the relationship between peripheral BDNF levels, TSH levels, and hippocampal volume in a series of antipsychotic-naïve FEP patients and their relation with symptomatology, the absence of a control group is also an important limitation. Another limitation is that we did not determine plasma T3 and T4 levels (only TSH). Finally, our results could have been influenced by cannabis and nicotine use (45, 46). However, we attempted to minimize this effect by adding these variables to the regression model. By contrast, the main strength of this study is the large sample size of antipsychotic-naïve FEP patients, a clinical population that is particularly difficult to recruit. Importantly, by excluding patients treated with antipsychotics, we were able to rule out the potential influence of those drugs on TSH and BDNF levels (13, 47), a potential confounding factor. Another important strength of the study is the novel approach used to understand the role of thyroid hormones in psychosis and hippocampal volume alterations in FEP patients.

## Conclusion

In the present study, one-third of antipsychotic-naïve FEP patients presented elevated TSH levels. Furthermore, TSH levels were inversely correlated with both peripheral BDNF concentration and hippocampal volume. These findings suggest that thyroid hormones could have a neuroprotective effect on the hippocampus of FEP patients, possibly through their effect on BDNF and through other cellular pathways. Further research is needed to better elucidate the potential causal association between alterations in thyroid hormone levels and the development of psychosis.

## Data availability statement

The original contributions presented in the study are included in the article/Supplementary material, further inquiries can be directed to the corresponding author.

## Ethics statement

The studies involving humans were approved by Research Ethics Committee with medications from the MAR Health Park. The studies were conducted in accordance with the local legislation and institutional requirements. The participants provided their written informed consent to participate in this study.

## Author contributions

AT: Writing – original draft, Writing – review & editing, Conceptualization, Formal analysis, Investigation, Methodology. LB-H: Formal analysis, Methodology, Supervision, Writing – review & editing. DB: Resources, Validation, Writing – review & editing. AnaM: Project administration, Writing – review & editing. KA: Project administration, Writing – review & editing. VP-S: Conceptualization, Funding acquisition, Writing – review & editing. AnnM: Writing – original draft, Writing – review & editing, Conceptualization, Investigation, Methodology, Supervision, Validation.

## Footnotes

^1^ http://dbm.neuro.uni-jena.de/cat/

^2^ http://surfer.nmr.mgh.harvard.edu

^3^ http://enigma.ini.usc.edu/protocols/imaging-protocols/

^4^ https://surfer.nmr.mgh.harvard.edu/fswiki/asegstats2table
