# Supplementary material for: Relationship between thyroid-stimulating hormone, BDNF levels, and hippocampal volume in antipsychotic-naïve first-episode psychosis patients
Source: Front Psychiatry. 2023 Dec 7;14:1301714. doi: 10.3389/fpsyt.2023.1301714 (PMC10734033; doi:10.3389/fpsyt.2023.1301714)
Supplement: Supplementary file 1 [file Data_Sheet_1.docx]

**ETEP Program (Study and Treatment Program for First Episode Psychosis)**

The Institute of Neuropsychiatry and Addictions–Parc de Salut Mar, Barcelona, Spain, has developed an FEP program with a set of coordinated inpatient and outpatient services that allows the efficient application of specific resources to all the patients in the program. The program started in 2008 and offers specific follow-up and immediate engagement after hospitalization, after an emergency department visit, or after referral by a general practitioner for a first episode of psychosis with a duration of no longer than two years. Other inclusion criteria were age between 18 and 35 years and estimated IQ higher than 80. Exclusion criteria were a medical history of neurological damage or head trauma and dependence on cocaine, stimulants, sedatives, or opioids (cannabis abuse or dependence was not an exclusion criterion).

All patients included in the study received psychiatric follow-up examinations according to general guidelines to ensure treatment with a second-generation antipsychotic drug at low to medium doses. When stabilization was achieved, patients were referred to the local outpatient service for treatment by a psychiatrist associated with the FEP program. Regular visits were scheduled once a week during the first month, once every two weeks in the second month, and once a month during further follow-up. More frequent visits were offered if needed. In accordance with U.S. and international recommendations (23,24) and studies of antipsychotic treatment discontinuation (25), patients who had experienced only a psychotic episode and who had been in clinical remission for more than one year (preferably two years) could discontinue antipsychotic medications but continue with follow-up appointments. All staff members of the FEP program met once a month. Patients reporting cannabis use were offered a specific psychological treatment for substance use, whereas patients who did not use cannabis were offered psychological assessment to help them cope with their illness. A structured program consisting of eight psychoeducation and informative sessions was offered to patients’ relatives to provide them with needed information about FEP. To reinforce social reintegration, patients who described difficulties resuming their academic or job activities were given the opportunity to attend one-hour weekly group sessions led by a social worker and a psychologist. These professionals identified each patient's handicaps in order to help them find the appropriate social, academic, or work support.
